# Fast discrimination of fragmentary images: the role of local optimal information

**DOI:** 10.3389/fnhum.2023.1049615

**Published:** 2023-02-08

**Authors:** Serena Castellotti, Ottavia D’Agostino, Maria Michela Del Viva

**Affiliations:** Department of Neurofarba, University of Florence, Florence, Italy

**Keywords:** fast vision, image recognition, information-optimal local features, visual saliency, image occlusion

## Abstract

In naturalistic conditions, objects in the scene may be partly occluded and the visual system has to recognize the whole image based on the little information contained in some visible fragments. Previous studies demonstrated that humans can successfully recognize severely occluded images, but the underlying mechanisms occurring in the early stages of visual processing are still poorly understood. The main objective of this work is to investigate the contribution of local information contained in a few visible fragments to image discrimination in fast vision. It has been already shown that a specific set of features, predicted by a constrained maximum-entropy model to be optimal carriers of information (optimal features), are used to build simplified early visual representations (primal sketch) that are sufficient for fast image discrimination. These features are also considered salient by the visual system and can guide visual attention when presented isolated in artificial stimuli. Here, we explore whether these local features also play a significant role in more natural settings, where all existing features are kept, but the overall available information is drastically reduced. Indeed, the task requires discrimination of naturalistic images based on a very brief presentation (25 ms) of a few small visible image fragments. In the main experiment, we reduced the possibility to perform the task based on global-luminance positional cues by presenting randomly inverted-contrast images, and we measured how much observers’ performance relies on the local features contained in the fragments or on global information. The size and the number of fragments were determined in two preliminary experiments. Results show that observers are very skilled in fast image discrimination, even when a drastic occlusion is applied. When observers cannot rely on the position of global-luminance information, the probability of correct discrimination increases when the visible fragments contain a high number of optimal features. These results suggest that such optimal local information contributes to the successful reconstruction of naturalistic images even in challenging conditions.

## Introduction

In the real world, humans are constantly exposed to partially occluded objects, which the visual system must analyze and recognize very quickly for survival purposes. Thus, in real scenes, the visual system copes with the recognition of incomplete images, whose mechanisms are still not completely understood. Many studies have demonstrated that humans can successfully recognize fragmented images (Brown and Koch, 2000; Murray et al., 2001; Johnson and Olshausen, 2005; Ullman et al., 2016; Tang et al., 2018), but most of them focus on the rules to solve the occlusion and on how the system fills the missing information. Instead, here we are not interested in understanding the mechanisms through which the visual system binds the fragments into a whole image. We rather focus on the identification of the most relevant fragments to be analyzed and on the extraction of salient local features within these fragments. Hence, we focus on the low-level stages of this process.

To explain the mechanisms of information selection, several models of visual search employ the concept of *saliency map*, a two-dimensional map that encodes the saliency of the objects in the visual scene (Itti et al., 1998). The saliency of an object depends on its physical properties (e.g., luminance contrast, contours’ orientation, etc.) and reflects the ability of that object to pop out in the visual scene. Each object in the scene competes for selection and only salient elements, those carrying the higher amount of information to the visual system, are chosen for further processing (Fecteau and Munoz, 2006). In this view, saliency operates very rapidly through bottom-up mechanisms: Salient features automatically attract our attention without any voluntary effort. From a neural perspective, it has been suggested that the primary visual cortex could provide a saliency map relying on specific processing of the local elements (Li, 2002). Visual saliency can be also influenced by contextual factors (Treisman and Gelade, 1980; Itti and Koch, 2001). Indeed, many studies related the selection of salient features to top-down mechanisms, by applying a spatially defined and feature-dependent weighting to the different feature maps (Wolfe et al., 1989).

The principles driving salience and the relative contribution of local (Li, 2002; Zhang et al., 2020) and global cues (Oliva and Schyns, 1997; Itti et al., 1998) are still under debate. Global and local information are related to spatial frequency: low spatial frequencies carry information about the global contrast distribution, whereas high spatial frequencies mainly provide fine information about local details (Blakemore and Campbell, 1969; Webster and de Valois, 1985; Boeschoten et al., 2005; Kauffmann et al., 2014). Nevertheless, several past studies have explored the mechanisms of fast vision at different scales and stimulus durations, finding that both coarse and fine spatial information are simultaneously used in fast image categorization (Oliva and Schyns, 1997; Schyns and Oliva, 1999).

In the present study, we hypothesize that the perception of incomplete images in fast vision partly starts from the extraction of some specific local high-frequency salient features contained in the visible image fragments. To identify salient features, we follow the principle that visual saliency may be based on the amount of local information (Shannon, 1948), as proposed by the constrained maximum-entropy model for early visual feature extraction (Del Viva et al., 2013). This model is founded on the need for a strong data reduction that must be operated by the visual system at an early stage, in order to optimize and speed up the reconstruction of visual images (Attneave, 1954; Barlow, 1961; Marr and Hildreth, 1980; Marr, 1982; Atick and Redlich, 1990; Atick, 1992; Olshausen and Field, 1996; Zhaoping, 2006). This is necessary given the huge amount of input data and the limited amount of neural resources (Attwell and Laughlin, 2001; Lennie, 2003; Echeverri, 2006; Del Viva and Punzi, 2014).

According to their model (Del Viva et al., 2013), in order to compress information and provide a saliency map of the visual scene, at an early stage the visual system selects only a very limited number of visual features for further processing. The features selected (*optimal* features) are those that produce in the output the largest amount of entropy allowed by the given computing limitations of this early stage filter (constrained maximum-entropy). The limitations considered by this model are the number of features transmitted and the output bandwidth (i.e., bandwidth and storage occupancy). Optimizing for entropy, together with the strict limitations on the computing resources, allows the system to completely determine the choice of the features from the statistical distribution of the input data. The authors proposed that only these features, which are *optimal* carriers of information, are *salient* in fast vision and used to represent visual images (*sketches*). All the other features that do not fulfill constrained maximum-entropy optimization criteria (*non-optimal* features) are considered not salient and are not transmitted to the following processing stages. Thus, unlike other models of early data compression based on redundancy reduction (Olshausen and Field, 1996), this approach leads to a huge loss of information. This is unavoidable given the limitations of the brain’s capacity, imposed by intrinsic energetic costs of neuronal activity and ecological limits to the number of neurons. The result is a fast, albeit heuristic, analysis of salient features in the visual scene. The implementation of the model on a set of black and white naturalistic images (i.e., depicting landscapes, animals, plants; Olmos and Kingdom, 2004), imposing strict limitations on the number of features and output bandwidth, led to the extraction of a set of *optimal* features, that, according to the model, are the only visual elements used to build the image *sketch*.

The reduction of input images to only two levels is a corollary of the central idea of compression by pattern filtering proposed by the model (Del Viva et al., 2013): The number of possible patterns, assumed to be a limited resource, increases exponentially with the number of allowed levels (that is 2n*N where n is the number of bits and N the number of pixels)—and so does the amount of computing needed to calculate them. Therefore, using a large number of gray levels in the model would be not only unpractical but also would defeat its very purpose of saving computational resources. For the same reason, the authors chose to implement the model by defining as a feature a 3 × 3-pixel image partition. Such a small size, corresponding to about 6 × 6 min of arc, also allows to target early visual processing stages. These are very likely the anatomical substrate of the hypothesized filter because data compression must be done very early in the visual stream to be effective. Although early visual structures comprise multiple cell types, with different receptive field sizes (Nassi and Callaway, 2009), here, for simplicity, a single small scale is considered. However, this small scale is consistent with receptive field sizes found in human V1, which are about 15′ in the fovea (Smith et al., 2001) and become progressively larger with eccentricity and through the hierarchy of visual areas (Zeki, 1978). At any rate, features of this size have been demonstrated to be still visually discernible by normal human subjects (Del Viva et al., 2013).

Sketches, obtained by retaining only *optimal* features in the digitized images, were presented very briefly to human observers and allowed very accurate discrimination of the original unfiltered images (higher than 80%), comparable even to that of showing the images themselves. Nevertheless, the loss of information was conspicuous: information contained in the sketches could reach 10% of the originals, compressing data by a factor of 40 (Del Viva et al., 2013).

The spatial structure of extracted features resembles the bar- and edge-like receptive fields found in primary visual cortices (Hubel and Wiesel, 1965), suggesting that these specific visual receptive fields represent the optimal way to transmit information in fast vision. In contrast, the features discarded by the model as *non-optimal* carriers of information have a uniform luminance structure (features with high bandwidth occupancy) or a “noisy” alternation of black and white pixels (features with high memory occupancy) (Del Viva et al., 2013).

In a further study, to assess the contribution of *optimal* local features to image discrimination, they were replaced with *non-optimal* features along the objects’ contours in the sketch. The disruption of optimal local cues in the sketches caused a decrease in image discriminability, despite preserving the global structure, suggesting that the fine structure of the image plays a crucial role in the discrimination (Del Viva et al., 2016).

Very recently, further studies showed that indeed these *optimal* features are considered salient even if they are presented in isolation without a global or semantic context (Castellotti et al., 2021), and they are able to automatically attract covert and overt attention (Castellotti et al., 2022).

Here we explore whether these specific local features still play an important role in more natural settings, where all existing features are kept (optimal and non-optimal), but the overall available information is drastically reduced. For this purpose, we created images where only a few fragments are shown, and the remaining parts are covered by a gray mask. In this way, we obtain visual stimuli with the same properties as the original images, in which the features are spatially and structurally unaltered, but the overall available information is reduced. To find the essential information needed to discriminate a visual scene, we pushed the visual system to its limits: the stimuli had very few visible parts and short durations. Specifically, participants had to covertly attend to a few briefly presented small fragments (or just one fragment) of binarized images (Del Viva et al., 2013) and then use them to discriminate the underlying image (target) from another (distractor).

Observers could solve this task by matching the position of black and white parts of the fragmented image and the target (global information), without the need to analyze the internal content of the fragments. If this were the case, we would expect the performance to depend on fragments contrast. On the other hand, performance could be related to the *optimal* information contained in the fragments, as predicted by the reference model. In this case, we would expect performance to depend on the number of local *optimal* features contained in the fragments. With multiple fragments covert attention could potentially be directed toward one of them; for this reason, we also measured discrimination by showing just a single fragment. This allowed us to correlate correct responses to the specific local information and contrast.

We then repeated the same discrimination task randomly inverting the contrast of the target and/or the distractor image. The purpose of this manipulation is to reduce the contribution of global information, given by the position of black/white large areas, and bring out the contribution of high-frequency components that could be masked by the prevalence of positional cues in original-contrast images.

Before testing our main experimental hypothesis in the Main experiment, we conducted two Preliminary experiments to test the limits for the discrimination of our fragmented digitized images, shown for a very short time. In these experiments, we probed the size and number of the fragments to be used in the Main experiment.

## Materials and methods

### Observers

Twenty young volunteers took part in this study. Ten observers (mean age = 25.3 ± 1.8 years) participated in Preliminary experiment 1, and five of them (mean age = 25.2 ± 1.8 years) also participated in Preliminary experiment 2. Ten other observers (mean age = 26.5 ± 2.9 years), all different from those of the preliminary experiments, participated in the Main experiment. All observers had normal or corrected to normal vision and no history of visual or neurological disorders. All participants gave written informed consent before the experiments. The study was conducted according to the guidelines of the Declaration of Helsinki and approved by the local ethics committee (“Commissione per l’Etica della Ricerca,” University of Florence, 7 July 2020, n. 111).

### Apparatus and set-up

The apparatus and set-up were the same for the Preliminary and the Main experiments. All stimuli were programed on an ACER computer running Windows 10 with Matlab 2018b, using the Psychophysics Toolbox extensions (Brainard, 1997; Pelli, 1997; Kleiner et al., 2007). The experiment was displayed on a gamma-corrected CRT Silicon Graphics monitor (1,152 × 864 pixels resolution, 38.5 × 29.5 cm, 120 Hz refresh rate), subtending 38.5 × 29.5 degree of visual angle at a 57 cm viewing distance. All experiments were carried out in a completely dark room. Participants’ manual responses were provided on a standard Dell keyboard.

### Procedure and stimuli

#### Preliminary experiment 1

**FIGURE 1:**
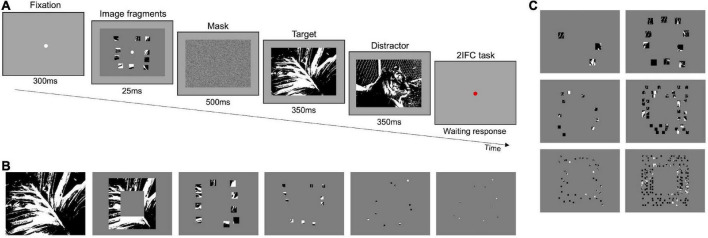
Preliminary experiments–Procedure and stimuli. **(A)** Representation of experimental paradigm. **(B)** Examples of stimuli used in Preliminary experiment 1. The first image is the control stimulus, the second is the “frame” stimulus, and the others show 10 fragments of decreasing size (in order: 7.5, 2, 0.47, and 0.12%), positioned within the frame. **(C)** Examples of stimuli used in Preliminary experiment 2. In the left column, fragments revealed 2% of the image area, and in the right column, fragments revealed 7.5% of the image area. Fragments’ size in the images of each column decreases by fifty percent going from top to bottom; whereas fragments in the same row have the same size but vary in number.

The experimental procedure is represented in Figure 1A. Each trial started with the presentation of a white fixation point (300 ms) on gray background (14 cd/m^2^) followed by the brief presentation (25 ms) of one stimulus in the center of the screen. Stimuli were composed of a certain number of image fragments of different sizes, resulting in a kind of “covered” image, revealing only small visible parts to the observer (see the paragraphs below for stimuli details). Immediately after, a mask appeared for 500 ms, followed by two black-white images sequentially presented for 350 ms each. One of the two images corresponded to the fragmented “covered” image (*target*), while the other (*distractor*) was randomly extracted from the set of images used (see the paragraphs below for image details). At each trial, the target was randomly presented in the first or the second interval. Images in the task were randomly displaced diagonally by 10 pixels, either to the top-left, top-right, bottom-left, or bottom-right, with respect to the position of the fragmented “covered” image. This spatial shift was purposedly introduced to avoid exact spatial matching between stimulus and target image. Observers were required to discriminate the target in a two-interval forced choice task (2IFC), by pressing a computer key.

Stimuli were prepared starting from 327 1-bit black and white renditions of naturalistic images, extracted from a public database (Olmos and Kingdom, 2004). Images’ size was 918 × 672 pixels, subtending 32.4 × 23.7° of visual angle at 57 cm. The luminance of white, black, and medium gray was 35, 1, and 12 cd/m^2^, respectively.

In Preliminary experiment 1, we measured discrimination as a function of the image’s visible area. We used the following stimulus configurations: the whole image as a control (100% visible area, see Figure 1B—first panel); a squared “frame” comprised between 4.8° and 8.8° of eccentricity (35.8% visible area, see Figure 1B—second panel); ten image fragments revealing different fractions of image area: 7.5% (size of all fragments 2.4 × 2.4°), 2% (size of all fragments 1.2 × 1.2°), 0.47% (size of all fragments 0.6 × 0.6°) and 0.12% (size of all fragments 0.3 × 0.3°; see Figure 1B—third to sixth panels, respectively). In these cases, the rest of the image was covered by uniform gray pixels. For further examples of stimuli, see Supplementary Figure 1. For each area, image fragments were randomly selected from all possible combinations satisfying the following conditions: (i) They had to be comprised in the 4.8–8.8° eccentricity frame (stimuli presented within this eccentricity are well visible even if observers have to maintain fixation in the center, as shown with other tasks; see for example, Larson and Loschky, 2009; Staugaard et al., 2016); (ii) they had to be evenly distributed within the frame three fragments on the top and bottom sides of the frame, and two fragments on each lateral side; (iii) they could not overlap with each other. The chosen frame width guarantees that criteria (ii) and (iii) are met. For each image, five different fragments’ configurations were created to minimize memory effects, for a total of 1,635 different stimuli for each area (see Supplementary Figure 2). A total of 3,000 trials per observer were run (300 trials for the control and frame conditions and 600 trials for each other condition). Each specific image configuration in each condition has been shown on average 1.2 times to each participant, preventing the association of a specific configuration of fragments to a target.

#### Preliminary experiment 2

Preliminary experiment 2 followed the same procedure as Preliminary experiment 1 (see Figure 1A). We measured discrimination as a function of the number of fragments of different sizes covering two different visible image areas (2 and 7.5%). The fragments were still positioned in the 4.8°−8.8° eccentricity frame. For 2% of the area we used: three 2.4 × 2.4° fragments (randomly distributed across the frame), ten 1.2 × 1.2° fragments (three fragments located on the top and bottom sides of the frame, and two fragments on the left and right sides), and 40 0.6 × 0.6° fragments (12 fragments located in the upper and lower side, and eight fragments in the left and right sides; see Figure 1C–left side panels, from top to bottom, respectively). For 7.5% of the area we used: 10 2.40 × 2.40° fragments (three fragments located on the top and bottom sides of the frame, and two fragments on the left and right sides), 40 1.2 × 1.2° fragments (12 fragments located on the top and bottom sides of the frame, and eight fragments on the left and right sides), and one 160 0.6 × 0.6° fragments (40 fragments located in the top, bottom, left, and right part of the image frame) (see Figure 1C–right side panels, from top to bottom, respectively). For further examples of stimuli, see Supplementary Figure 3. For each image, five different fragments’ configurations were created, for a total of 1,635 different stimuli for each area (see Supplementary Figure 2). A total of 3,600 trials per observer were run (600 trials for each condition). Each specific image configuration in each condition has been shown on average 1.1 times to each participant.

#### Main experiment

**FIGURE 2:**
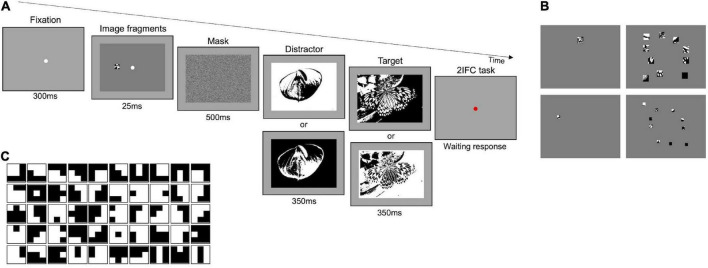
Main experiment–Procedure and stimuli. **(A)** Representation of experimental paradigm. The example illustrated in the upper row shows a trial where the distractor is presented with inverted contrast and the target with its original. The two panels below target and distractor indicate that in some trials either one or both of them can have a contrast inverted with respect to those shown above. **(B)** Examples of stimuli. Fragments in the images of each column are the same number but their size decreases by 50 percent from top to bottom; fragments in the images of each row have the same size but vary in number (1 or 10). **(C)** Set of *optimal* features. Set of 50 3 × 3-pixels features extracted by applying the constrained maximum entropy model to black and white images (Del Viva et al., 2013).

The Main experiment follows the same procedure (2IFC) and used the same set of images (Olmos and Kingdom, 2004) as those of the Preliminary experiments 1 and 2, but participants were engaged in two different tasks: a task with original-contrast images and a task with randomly inverted-contrast images. In the first task, both the target and the distractor were digitized versions of the original images (as in Figure 1A). In the second task, in some randomly selected trials, the target and/or the distractor had their contrast inverted with respect to their original version (Figure 2A). Therefore, in some trials both the target and the distractor could be presented with their original or inverted contrast, while, in other trials, only one of them could have inverted contrast. With this manipulation, we aim at reducing the probability of solving the task by matching the position of black and white spots in the fragments to those in the images (see Supplementary Figure 4). Each image has been presented to each participant on average 37.7 times, either as a target or distractor.

In both tasks, the same conditions were tested. Stimuli consisted of one or ten fragments (see Figure 2B—first and second column, respectively) with different sizes: 2.4 × 2.4° and 1.2 × 1.2° (see Figure 2B–first and second row, respectively). The total area revealed by these fragments was 0.2 and 0.75% with one fragment, 2 and 7.5% with ten fragments. The characteristics of the stimuli (luminance, fragments distribution, and eccentricity) were the same as those used for Preliminary experiments 1 and 2. For further examples of stimuli, see Supplementary Figure 5. In the condition with 10 fragments, for each image, five different fragments’ configurations were created, for a total of 1,635 different stimuli for each area (see Supplementary Figure 2). In the condition with 1 fragment four/five different configurations were created, for a total of 1,144 and 1,253 different stimuli for 0.2 and 0.75% area, respectively (see Supplementary Figure 6). In the Main experiment, each observer performed 2,400 trials in total: 1,200 trials in the task with original-contrast images (300 trials for each stimulus condition), and 1,200 trials in the task with randomly inverted-contrast images (300 trials for each stimulus condition). Each specific image configuration in each condition has been shown on average 1.1 times to each participant.

### Data processing and statistical analysis

In all experiments, we measured the percentage of correct responses of each observer in each condition of visible area.

In Preliminary experiments 1 and 2, non-parametric one-way repeated-measures ANOVAs (Friedman’s tests) with Conover *post hoc* comparisons (Bonferroni correction) were used to test differences between averaged performances across conditions. In Preliminary experiment 1, we also performed a one-sample Wilcoxon signed-rank test to assess whether the averaged performance in the condition with the smallest visible image area was still above the chance level (i.e., statistically different from 50%).

In the Main experiment, non-parametric two-way repeated-measures ANOVAs (Durbin tests) with Conover *post hoc* comparisons (Bonferroni correction) were used to test differences between average participants’ performances in each condition of visible area in the original vs. inverted contrast tasks.

In addition, all observers’ data were pooled together to calculate the performance as a function of fragments’ contrast and signal-to-noise ratio (SNR) in each condition of visible area.

We calculated the Weber contrast of the fragment as follows: We first averaged the pixel values within the fragment (black = 0, white = 255), then this averaged value was subtracted from the background value (gray = 127), and finally the absolute value of the ratio between the result of the subtraction and the background was calculated. In the stimuli containing ten fragments, the average contrast of the fragments was considered. The performance was then analyzed as a function of Weber contrast (bins of 0.2 each).

To quantify the *saliency* of each fragment we calculated the signal-to-noise ratio (SNR), that is the number of *optimal* features, predicted salient by the reference model, over the total number of features. Specifically, we considered a set of 50 *optimal* features, 3 × 3 pixel large (see Figure 2C), each subtending ∼0.1 × 0.1° of visual angle (about 12 c/deg spatial frequency). This specific set of *optimal* features has been proven to be salient for humans in previous works (Del Viva et al., 2013; Castellotti et al., 2022, 2021). In the stimuli containing ten fragments, the average SNR of the fragments was considered. The performance was then analyzed as a function of SNR (bins of 0.05 each).

For each SNR bin, we calculated the average contrast of fragments with the standard error. The Pearson linear-correlation coefficient between SNR and contrast was then calculated.

Given the strong correlation between fragments’ contrast and SNR, to quantify their relative contribution to the performance, we created a new variable by subtracting, in each trial, the standardized values from each other (SNR—contrast).

Data from all conditions of visible area (7.5, 2, 0.75, and 0.2%) were pooled together and GLMMs with a binomial error structure were performed. In the task with original contrast images, the model included three fixed factors: (i) SNR-contrast difference (standardized); (ii) target order presentation, to test whether the performance depended on the fact that the target was in the first vs. second interval; (iii) image repetition number (i.e., the frequency of occurrence of each image as target or distractor), to control for possible effects of visual memory. Participants and stimuli were included as random effects. In the task with randomly inverted-contrast images an additional fixed factor was included: (iiii) target contrast inversion, to test whether the performance changed in the trials where the target was presented with original or inverted contrast.

We then compared (*z*-tests) the probability of correct responses (with binomial standard deviations) between the task with original-contrast images and the one with random contrast inversion. This was done separately for the trials where the target had original contrast and for those where the target had inverted contrast.

Finally, a GLMM was run in the task with randomly contrast-inverted images including only the trials where the target had original contrast.

## Results

### Preliminary experiment 1

**FIGURE 3:**
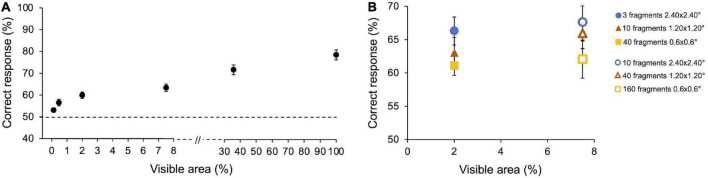
Preliminary experiments–Results. **(A)** Performance as a function of images’ visible area. Performance averaged across participants (*n* = 10) with SE. Observers performed 3,000 trials in total (300 trials for 100% area and frame conditions and 600 trials for each other areas condition). **(B)** Performance as a function of the number and size of image fragments. Performance averaged across participants (*n* = 5) with SE. Two image areas have been tested: Filled symbols indicate fragments revealing 2% of the area; empty symbols indicate fragments revealing 7.5% of the area. Symbols with the same shape indicate a different number of fragments of the same size. Observers performed 3,600 trials in total (600 trials for each condition).

Average performance in Preliminary experiment 1 (*n* = 10) is reported in Figure 3A. As expected, the percentage of correct responses increases with the size of the image fragments (i.e., the amount of visible area of the image). On average, observers’ performance ranges from 55% for the smallest visible area to 83% when the full image is shown (100% area). Particularly, observers gave 54.5 ± 1.03% (SE) correct responses at 0.12% of visible area, 58.3 ± 1.7% at 0.47%, 62 ± 1.6% at 2%, 65.8 ± 1.9% at 7.5%, 75.4 ± 2.4% at 35.8, 83.1 ± 2.5% at 100%. Friedman’s test showed a main effect of the visible area [χ^2^(5) = 45.3, *p* < 0.001, *W* = 0.46]. All Conover *post hoc* comparisons (Bonferroni correction) are reported in Supplementary Table 1.

The average performance obtained by showing the smallest image area also resulted statistically different from 50% [*Z* (9) = 55, *p* = 0.002], showing that observers are able to discriminate an image based on very little information.

### Preliminary experiment 2

In Preliminary experiment 2 (*n* = 5), we compared the observers’ performance when the same amount of image area is revealed by showing a different number of fragments of different sizes. Performances are reported in Figure 3B. For both areas tested (2 and 7.5%), the percentage of correct responses tends to be greater with few big fragments than with more small fragments, even if none of the results are statistically significant. When the size of the patches remains constant but their number increases, thus revealing a bigger amount of image area to the observers, the performance slightly increases in all conditions, although not significantly. Specifically, when the percentage of the revealed image area is 2%, average performance is 66.3 ± 2.1% (SE) with three 2.40 × 2.40° fragments, 63.03 ± 2.3% with ten 1.20 × 1.20° fragments, and 61.1 ± 1.5% with forty 0.6 × 0.6° fragments. When the percentage of the revealed image area is 7.5%, average performance is 67.6 ± 2.8% with ten 2.40 × 2.40° fragments, 65.9 ± 2.2% with forty 1.20 × 1.20° fragments, and 62.1 ± 2.8% with one hundred and sixty 0.6 × 0.6° fragments.

### Main experiment

**FIGURE 4:**
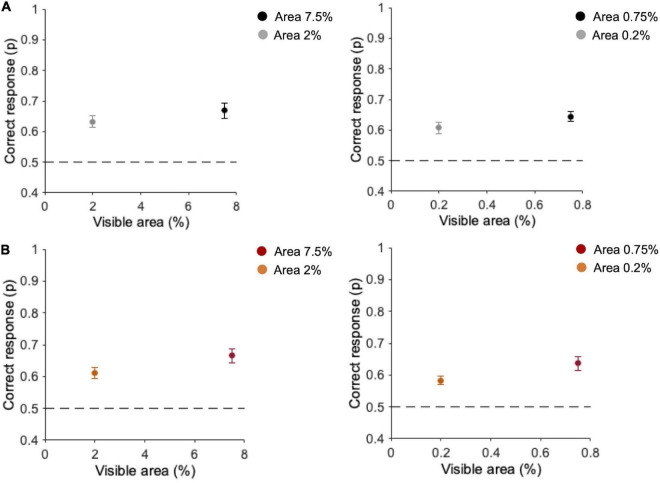
Performance for different areas and number of fragments. **(A)** Task with original-contrast images. **(B)** Task with randomly inverted-contrast images. Left panels: average performance (*n* = 10) for ten fragments (2 and 7.5% of area); Right panels: average performance (*n* = 10) for one fragment (0.2 and 0.75% of area). Errors are SE across participants. Observers performed 2,400 trials in total (300 trials for each condition).

In the Main experiment (*n* = 10), we first analyzed the percentage of correct discrimination in the two tasks. In the task with original-contrast images (Figure 4A), when ten fragments are presented, observers’ discrimination is 63.3 ± 1.8% (SE) for 2% area and 68.8 ± 2.5% for 7.5% area (Figure 4A—left panel). With one single fragment, the average observers’ performance is 60.7 ± 2% at 0.2% area and 64.3 ± 1.6% at 0.75% area (Figure 4A—right panel). In the task with randomly inverted-contrast images (Figure 4B), with ten fragments discrimination performance is 61.1 ± 1.8% at 2% area and 66.7 ± 2.2% at 7.5% area (see Figure 4B—left panel). With one single fragment, the average observers’ performance is 58.3 ± 1.3% at 0.2% area and 63.6 ± 2.1% at 0.75% area (Figure 4B—right panel). Durbin test between performances with original- vs. randomly inverted-contrast images confirmed the effect of visible area [χ^2^(1) = 9.2, *p* = 0.002, *W* = −20] but no statistical differences emerged across the two tasks [χ^2^(1) = 0.2, *p* = 0.61]. This suggests that, even if in some trials of this task there is no correspondence between the contrast of the fragments and that of the target image, the overall performance is comparable to that obtained in the task with original-contrast images.

We then investigated to what extent the performance depended on the *saliency* of the local high-frequency features contained in the fragments presented (as predicted by the constrained maximum-entropy model), or on the global luminance information (Weber *contrast*). Firstly, we calculated performance as a function of SNR and contrast separately. In the task with original contrast images, performance does not depend on SNR, and it does not seem to be related to fragments’ contrast as well, although there is a tendency to increase with contrast with multiple fragments (Supplementary Figures 7A–B). Instead, in the task with randomly inverted-contrast images, the performance is higher for lower contrasts and decreases for higher contrasts, whereas it increases from lower to higher SNR (Supplementary Figures 7C–D).

**FIGURE 5:**
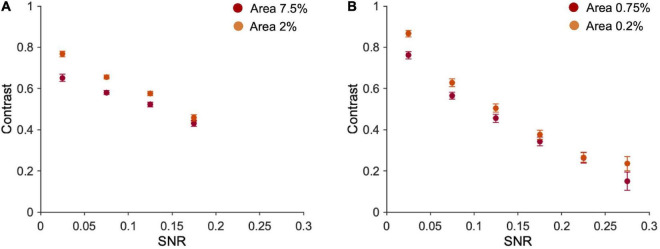
Fragments’ contrast vs. their SNR. **(A)** Average contrast of ten fragments vs. their average SNR (bins of 0.05 each). Number of occurrences in each bin (from the first to the last bin), 7.5% area: 345, 1,380,783,491; 2% area: 497,1202,857,444. **(B)** Average contrast vs. averaged SNR of one fragment (bins of 0.05 each). Error bars are standard errors. Number of occurrences in each bin (from the first to the last bin) = 0.75% area: 834,873,586,425,235,47; 2% area: 748,764,620,440, 308,120.

Note however that fragments’ contrast and SNR are negatively correlated (Figure 5; 7.5% area: *r* = −0.63, *p* < 0.001; 2% area: *r* = −0.72, *p* < 0.001; 0.75% area: *r* = −0.60, *p* < 0.001; 0.2% area: *r* = −0.69, *p* < 0.001). This correlation depends on the nature of the fragments and the way the two variables have been calculated: fragments with lower contrast are those containing a higher number of optimal features (high SNR), because high SNR reflects into a textured stimulus, and averaging alternations of many black and white pixels, leads to low Weber contrast. On the other end, fragments with higher contrast are those with large black/white parts and therefore contain a few optimal features (see Supplementary Figure 8). Note that the maximum SNR in the case of ten fragments (0.2) is lower than for one fragment (0.3) because, being the contrast mediated across ten different parts, the probability of having fragments with large black and white parts (and consequently low SNR) is higher.

Since the correlations between SNR and contrast are quite high, in the following analysis we used the difference between standardized SNR and contrast, instead of considering them as two separate variables. In this way, the contributions of SNR and contrast to the performance can be separated. Moreover, in a 2IFC task, the order of target presentation might affect the performance, as well as the frequency of occurrence of each image: repeated presentations of the same image as target or distractor might induce visual learning of the images. For the task with original contrast images, we then performed a GLMM with three fixed factors: SNR-contrast difference (standardized), target order presentation, and image repetition number. Participants and stimuli were included as random effects. The GLMM reveals no effect of the difference between standardized SNR and contrasts [χ^2^(1) = 0.24, *p* = 0.62], but a main effect of order [χ^2^(1) = 9.1, *p* = 0.002] and image repetition number [χ^2^(1) = 19.2, *p* < 0.001] emerges. Contrasts and marginals means are reported in Supplementary Table 2.

**FIGURE 6:**
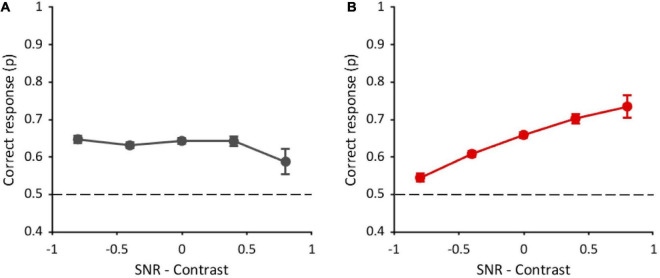
Performance as a function of the difference between standardized SNR and contrast. **(A)** Task with original-contrast images. **(B)** Task with randomly inverted-contrast images. Data from all observers (*n* = 10) and all conditions of visible area (7.5, 2, 0.75, and 0.2%) are pooled together. Errors are binomial standard deviation. Dashed lines represent chance level.

Overall, these results indicate that, in the task with original-contrast images, the performance does not depend on SNR (as shown in Figure 6A), and it does not seem to be related to fragments’ contrast either (although there is a tendency to increase with contrast with multiple fragments; see Supplementary Figures 7A–B). Given our hypotheses, we argue that in this condition observers do not rely on local cues and possibly use the position of black and white spots to solve the task. This hypothesis seems to be further supported by the fact that the performance is higher when the target is presented in the first interval of the 2IFC. Indeed, the match between the fragments and the corresponding image is easier if the target is temporally closer and its presentation is not interspersed with the appearance of the distractor.

We then performed the same analysis in the task with randomly inverted-contrast images (Figure 6B), used to reduce the contribution of positional global cues and to bring out the contribution of high-frequency optimal features (see Supplementary Figure 4). In this task, an additional factor was included in the GLMM. Considering all visible area conditions (12,000 trials in total), due to the random nature of inversion, the target contrast alone was inverted in 24.5% of trials, the distractor contrast alone was inverted in 25.2% of trials, the contrasts of both the target and distractor were inverted in 22.8% of trials, and the contrasts of both target and distractor were kept original in 27.4% of trials. In principle, these different target conditions could affect performance. The GLMM analysis was thus performed with four fixed factors (standardized SNR-contrast difference, target order presentation, image repetition number, and target contrast inversion) and two random effects: participants and stimuli. Contrasts and marginal means are reported in Supplementary Table 3. The analysis shows a significant effect of SNR-contrast difference [χ^2^(1) = 128.4, *p* < 0.001] on performance. Indeed, performance increases with this difference (Figure 6B), suggesting that SNR prevails over contrast in driving the performance. The target order factor is instead not statistically significant [χ^2^(1) = 0.07, *p* = 0.78], meaning that the performance does not change whether the target image is shown in the first or the second interval of the 2IFC task. These results confirm further our hypothesis that, in this condition, participants change their strategy: They do not rely on positional cues anymore, but rather they use local information, therefore target order does not affect the performance. Again, the analysis reveals an effect of the image repetition number [χ^2^(1) = 36.2, *p* < 0.001]. The target contrast inversion factor is also statistically significant [χ^2^(3) = 45.5, *p* < 0.001]. Indeed, the performance with original-contrast target (65 ± 0.006%) is higher than with inverted-contrast target (60 ± 0.006%).

Interestingly, the performance in the task with randomly inverted-contrast images in the trials with original-contrast target is also higher than that obtained in the task with original-contrast images (63 ± 0.004%; *z* = 2, *p* = 0.04), although these two conditions are exactly the same.

The GLMM analysis, including only the trials with original-contrast target of the task with randomly inverted-contrast images, reveals a main effect of the difference between SNR–contrast [χ^2^(1) = 33.9, *p* < 0.001; see Supplementary Figure 9], and of image repetition number [χ^2^(1) = 18.4, *p* < 0.001], but there is no effect of target presentation order [χ^2^(1) = 0.31, *p* = 0.58]. Contrasts and marginals means are reported in Supplementary Table 4. These results are compatible with those found when considering all trials, independently of target contrast inversion (see Figure 6B). On the other end, these results are different from those found in the task with original-contrast images (see Figure 6A), although these two conditions are exactly the same. See the Discussion section for the interpretation of these results.

## Discussion

In the present work, we investigated the visual system’s ability to quickly discriminate a scene, based on the salience of high-frequency local visual features.

Over the years, different studies have argued that the selection of relevant local elements is based on the simultaneous processing of different visual properties at multiple spatial scales, then combined into a single saliency-map (Itti et al., 1998; Itti and Koch, 2001; Torralba, 2003). However, these models do not consider the amount of computing power required by each parallel process. Our reference model, instead, takes into account the system’s computational costs. Considering the finest spatial scale as the most computationally demanding part of the processing and the need for fast analysis, the model applies a lossy data compression algorithm to images at a fine spatial scale (Del Viva et al., 2013). The result of this process is the extraction of a limited number of informative high-frequency visual features, that are used for fast image discrimination and to drive bottom-up attention (Castellotti et al., 2022, 2021).

Before investigating their role in fast discrimination of fragmented images, often presented to the visual system due to occlusions, we showed that observers can discriminate an image presented only for 25 ms even when it’s almost totally occluded. As expected, correct discrimination increases with the visible area, but is still possible with very little information (0.12%). These findings confirm that humans are very skilled in fast visual discrimination, as already broadly demonstrated (for a review, see Serre et al., 2007). Note however that we pushed the visual system’s capacity to its limit, by showing images for the minimum duration necessary for a visual stimulus to reach primary cortical visual structures (Grill-Spector et al., 2000; Kirchner and Thorpe, 2006) and by using a paradigm that is known to be challenging for the observers (i.e., 2IFC tasks lead to higher error than 2AFC, Jäkel and Wichmann, 2006); This might explain why observers did not reach top performance even when the full image is displayed (100% area). Despite this, the minimal percentage of visible area needed to perform the task is much lower (0.12%) than that found in previous studies. For example, Tang et al. (2018) conducted an experiment similar to ours, with occluded or partially visible images presented for different durations, finding that in 25 ms observers robustly recognized objects when they were rendered <15% visible (Tang et al., 2018). The higher performance with a smaller visible area found here could be explained by the different tasks involved: their participants had to choose the right association between the occluded content and five different label options, while ours discriminate between two images.

We also investigated which factors mostly influence the correct discrimination of occluded pictures. That is, we studied whether, with the same amount of visible area, discrimination depends more on the number of visible fragments or on their size. Results show a slight (not significant) preference for a few large fragments, rather than for many small parts. This is somewhat unexpected. However, some have hypothesized that perceptual systems suffer from overload, so the higher the perceptual load of current information, the lower the ability to perceive additional information (Greene et al., 2017). Here a low number of fragments could produce a lower cognitive load (Xing, 2007; Nejati, 2021), hence better performance.

In the Main experiment, we investigated the role of the high-frequency model-predicted *optimal* features in fragmented image discrimination by quantifying the saliency of the fragments as the ratio of *optimal* features over the total number of features they contain. That is, the question is whether observers focus on the local internal content of the fragments and use embedded optimal features to discriminate the target, or whether they covertly attend to the global contrast information (low frequency). Indeed, since we use black and white stimuli and a 2IFC discrimination task, observers could simply solve the task by matching the position of black and white parts of the fragmented image and the target, without the need to analyze the internal content of the patches.

When low frequencies can be used to perform the task (original contrast), the performance does not depend on the number of optimal features contained in the fragments, rather there is a slight tendency to increase with fragments contrast (particularly when ten fragments are shown). These results suggest that in this condition observers do not use local information but possibly use the fragments’ global luminance distribution. This hypothesis is further supported by the evidence that, only in the task with original-contrast images, the performance increases if the target is shown in the first interval of the 2IFC task. Indeed, we can assume that the match between the position of the black and white parts of the fragmented image and the target is easier if the latter is temporally closer to the stimulus and there is no other image before it.

A higher performance in the task with original-contrast images than in the task with random contrast inversion would be expected, since, in the former, positional cues can always be used. The fact that the performances in the two tasks are similar suggests that, when the contribution of global information is decreased (random inversion of contrast), observers rely on a different kind of information to discriminate the scene. In fact, we found that the probability of correct discrimination increases with the number of optimal features in the fragments, both with one and ten fragments, indicating that observers’ responses in the task with random inversion of contrast are based on the local content of the fragments. This change of strategy is further supported by the evidence that, in this condition, the performance does not depend on the target order of presentation. We argue that, since observers do not base their choice on positional cues, it doesn’t matter anymore if the target is presented in the first or in the second interval.

In the task with randomly inverted-contrast images in some trials the target still has the original contrast, therefore the global luminance structure of the fragments could still drive discrimination. Interestingly, considering only these specific trials, the performance is even higher than that obtained in the task where only original-contrast images are used, even though the two conditions are exactly the same. More importantly, correct responses depend on the number of optimal features in the fragments, and they are independent of target order, unlike in the task with original-contrast images. These results confirm that the contrast manipulation we applied in this task can change the observers’ strategy. In this condition, participants seem to use both global and local information reaching a higher performance than when they rely only on global information. We, therefore, conclude that when less global information is available, local information plays a crucial role.

Note that the set of optimal features comprises spatial structures with both contrast polarities; this could explain why the inversion of contrast does not affect discrimination based on local information. The insensitivity to contrast inversion (Baylis and Driver, 2001; Niell and Stryker, 2008) found in V1 complex cells, together with the similarity of spatial structure between model-predicted optimal features and the bar and edge-like V1 receptive fields (Hubel and Wiesel, 1965), strongly suggests that these cells represent the optimal way to transmit information in fast vision. This also highlights the strong predictive power of the constrained maximum-entropy model.

Overall, our findings suggest that local and global analyses interact in fast image processing and that the contribution of the high-frequency optimal features significantly emerges when the visual system is tested in very challenging conditions. This means that local information, when derived from maximum-entropy optimization criteria coupled with strict computational limitations, allows fast image discrimination even when the information about the scene is drastically reduced.

This fast local extraction of salient features must be operated very early in the visual pathway (Li, 2002; Del Viva et al., 2013), and integrated into a global percept at later visual stages. Indeed, in real scenes the visual system “goes beyond the information given” in a local region (Meng and Potter, 2008) and fills in the missing information of occluded images by binding the visible image fragments (Bruno et al., 1997; Johnson and Olshausen, 2005; Meng and Potter, 2008). Also, in daily life, the *a priori* knowledge of the objects helps the visual system in image recognition (Pinto et al., 2015; Stein and Peelen, 2015). Long-term memory, which is capable of storing a massive number of details from the images (Brady et al., 2008), contributes as well. Visual learning effects also occurred in our experiment, since the performance is affected by repeated presentations of the same image. This indicates that participants might have become acquainted with image details, revealing that there are some memory effects at play. Studies of the mechanisms of recognition of incomplete images have also developed information-statistical approaches, the concepts of the extraction of the signal from noise, and models of matched filtration (for a review, see Shelepin et al., 2009).

To conclude, our study confirm that local visual saliency can be determined by the amount of information that local features carry about the visual scene weighed with their processing costs for the system, as predicted by the reference model (Del Viva et al., 2013). What cannot be ignored is the fact that while viewing a scene, humans make eye movements several times per second. Considering these results as a starting point for further studies, it would be interesting to investigate whether saccades are directed toward the most informative areas, represented by the optimal features predicted by our reference model, to reconstruct the image.

## Data availability statement

The datasets presented in this study can be found in online repositories. The names of the repository/repositories and accession number(s) can be found below: https://doi.org/10.5281/zenodo.7096390.

## Ethics statement

The studies involving human participants were reviewed and approved by the Local Ethics Committee: “Commissione per l’Etica della Ricerca,” University of Florence, 7 July 2020, no. 111. The patients/participants provided their written informed consent to participate in this study.

## Author contributions

SC and OD participated in the experiment programing, data collection, statistical analyses, and manuscript writing and review. MMDV participated in the project ideation, statistical analyses, and manuscript writing and review. All authors contributed to the article and approved the submitted version.
