# Supplementary material for: Fast discrimination of fragmentary images: the role of local optimal information
Source: Front Hum Neurosci. 2023 Feb 8;17:1049615. doi: 10.3389/fnhum.2023.1049615 (PMC9945129; doi:10.3389/fnhum.2023.1049615)
Supplement: Supplementary file 1 [file Data_Sheet_1.pdf]

SUPPLEMENTARY MATERIAL

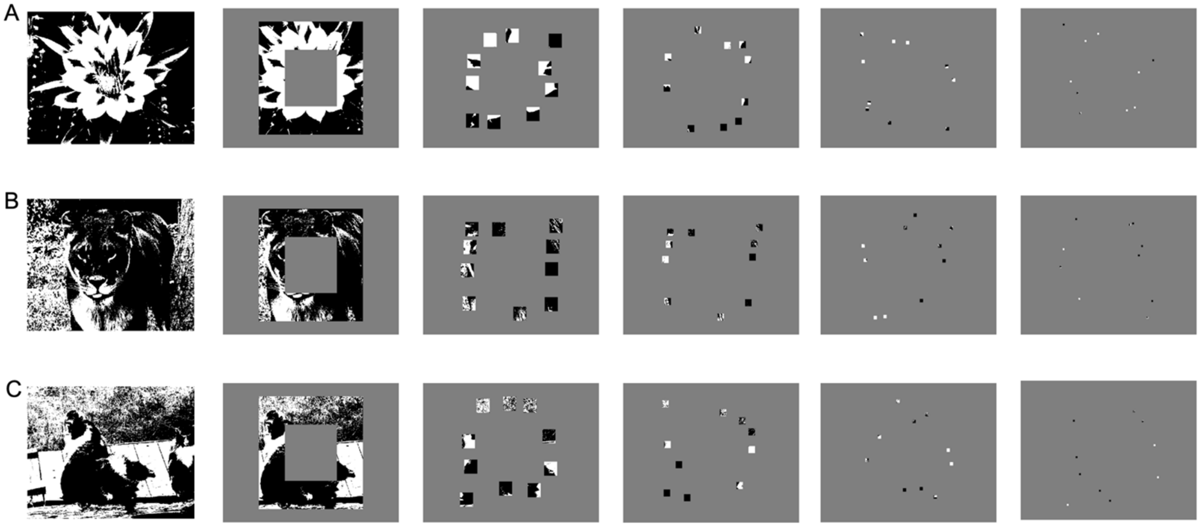

Supplementary Figure 1. (A-C) Examples of stimuli used in the first preliminary experiment. From left to right: images with 100% to 0.12% visible area.

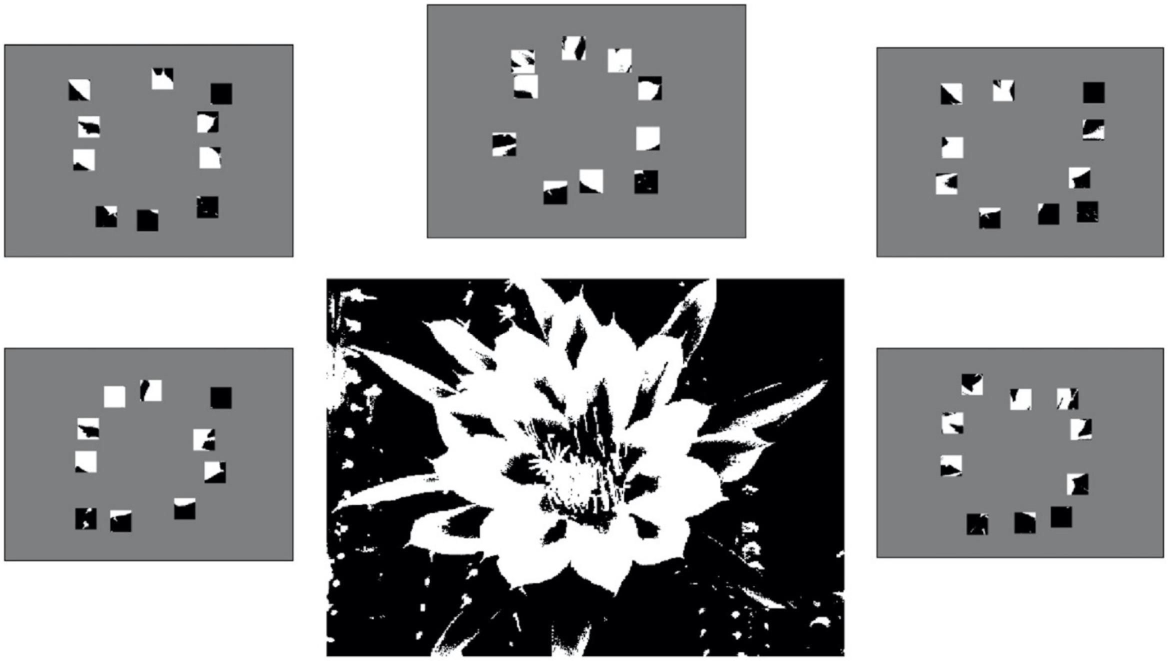

Supplementary Figure 2. Examples of different fragments' configurations for a specific image. Five different stimuli with ten 2.40°x2.40° fragments, covering 7.5% of the image area.

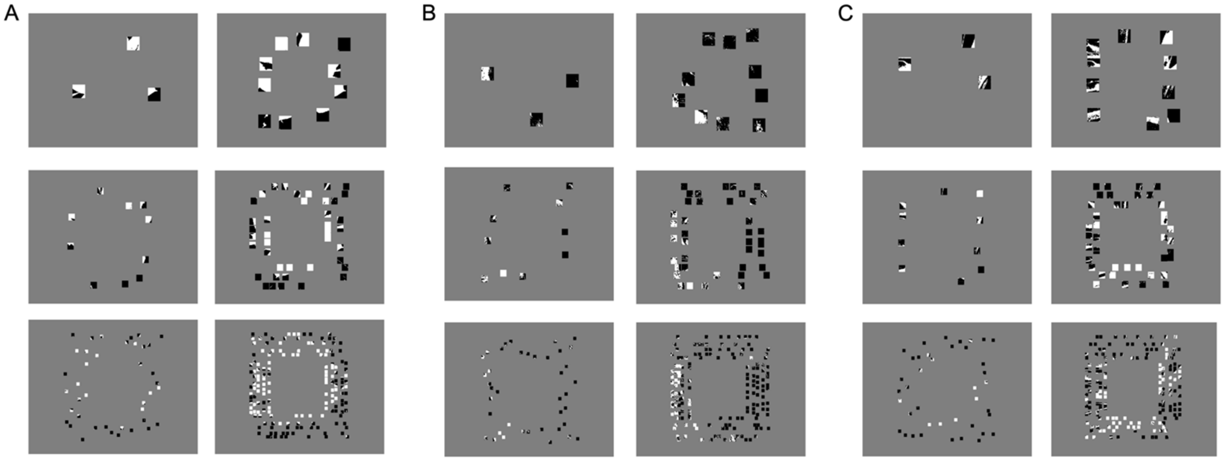

Supplementary Figure 3. (A-C) Examples of stimuli used in the second preliminary experiment. In the left column fragments revealed 2% of the image area, and in the right column, fragments revealed 7.5% of the image area. Fragments' size in the images of each column decreases by fifty percent going from top to bottom; whereas fragments in the same row have the same size but vary in number.

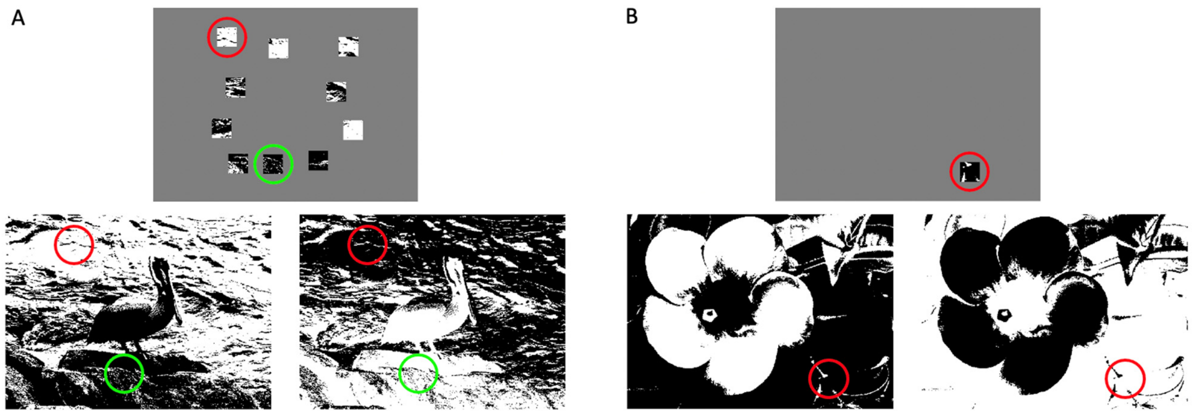

Supplementary Figure 4. Examples of tasks with original and inverted contrast images. The upper panels show two examples of fragmented images. The lower left-side panels show the original-contrast images; the lower right-side panels show inverted-contrast images. In these examples, the position of fragments with large black or white parts (red/green circles) can be easily matched in the original contrast images. **(A) Ten fragments (7.5% area).** In the original-contrast image (left-side panel), observers can match the position of the almost all-white fragment presented in the upper-left part of the image and that of the almost all-black fragment presented in the lower part to discriminate the target. Instead, with the inverted-contrast image (right-side panel), this positional match cannot be done. **(B) One fragment (0.75% area).** In the original-contrast image (left-side panel), observers can match the position of the almost all-black fragment presented in the lower-right part of the image to discriminate the target. In the inverted-contrast image (right-side panel), observers cannot find the black spot in the lower-right part of the target.

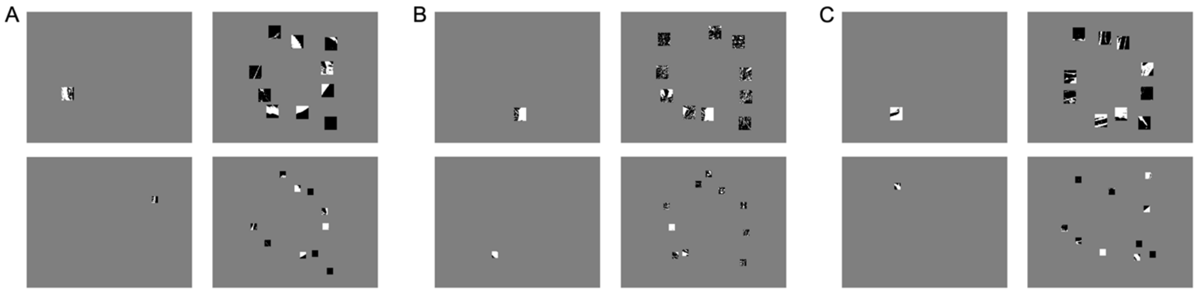

Supplementary Figure 5. (A-C) Examples of stimuli used in the main experiment. Fragments in the images of each column have the same number but their size decreases by fifty percent from top to bottom; fragments in the images of each row have the same size but vary in number (one or ten).

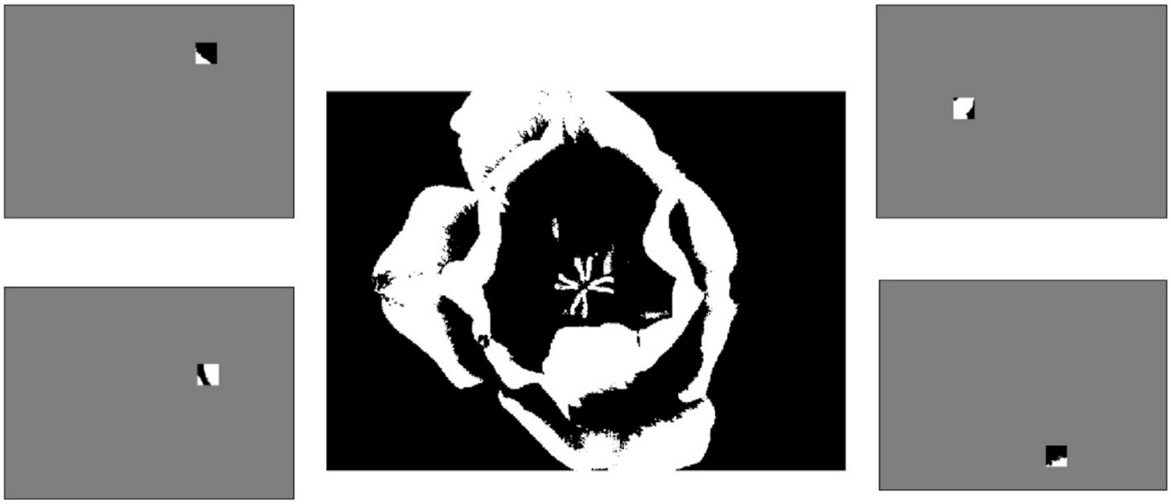

Supplementary Figure 6. Examples of different fragments' configurations for a specific image. Four different stimuli with one 2.40°x2.40° fragment, covering 0.75% of the image area.

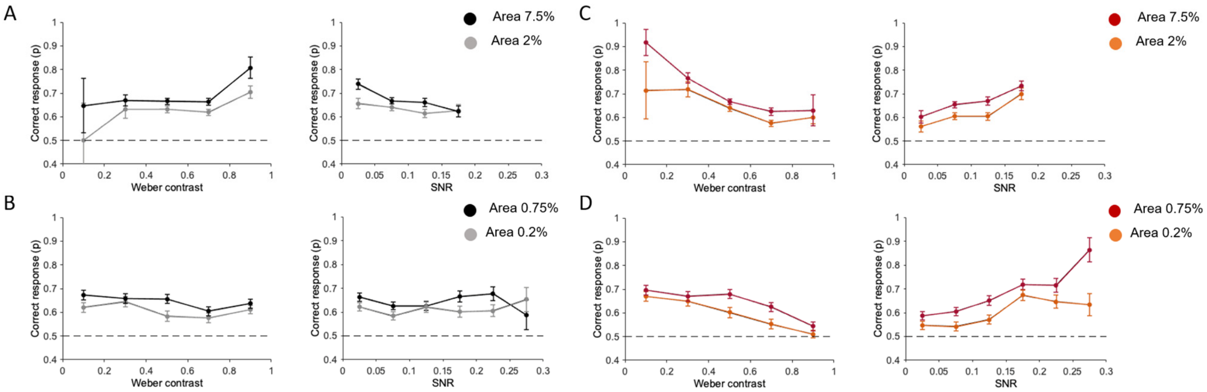

Supplementary Figure 7. Performance as a function of fragments' Weber contrast and SNR. (A) Task with original-contrast images with ten fragments. **(B)** Task with original-contrast images with one fragment. **(C)** Task with randomly inverted-contrast images with ten fragments. **(D)** Task

with randomly inverted-contrast images with one fragment. Error bars are binomial standard deviations. Observers performed 1200 trials in total (300 trials for each stimulus condition).

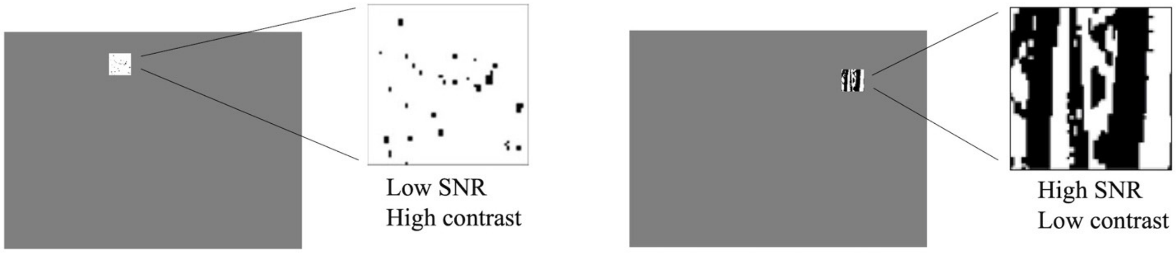

Supplementary Figure 8. Relationship between contrast and SNR in a fragment. Left panel: example of an almost totally white fragment, which has high contrast with respect to the background, but contains few *optimal* features (low SNR). Right panel: example of a fragment containing a textured internal structure, which has a contrast similar to the background (low contrast) but contains a high number of *optimal* features (high SNR).

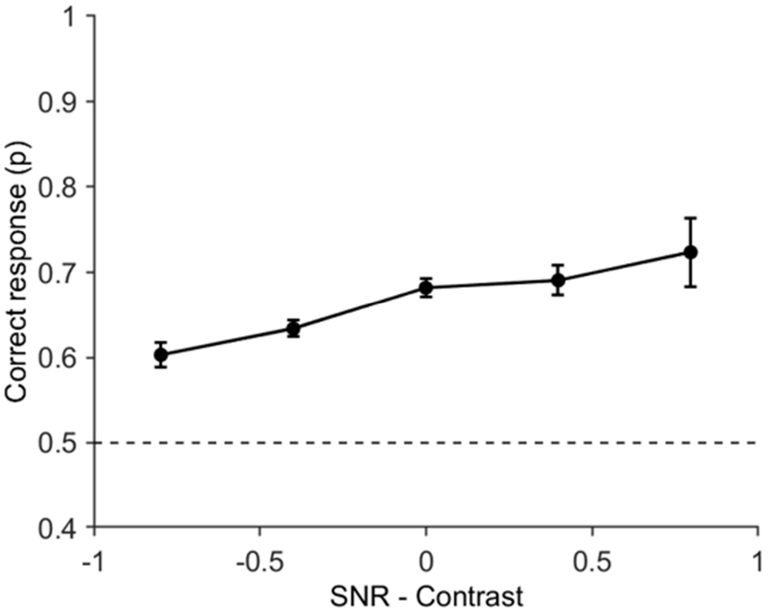

Supplementary Figure 9. Task with randomly inverted-contrast images - Performance as a function of the difference between standardized SNR and contrast in trials with original-contrast targets. In the graph, data from all participants (n = 10) and area conditions (7.5%, 2%, 0.75%, 0.2%) are pooled together (bins of 0.2 each - binomial standard deviations), considering only trials where the target was not contrast-inverted (6311 trials in total).

Supplementary Table 1. Preliminary experiment 1 (n = 10) - Conover's post-hoc comparisons (Bonferroni correction) across average performances for each area condition (3000 trials in total per observer: 300 trials for 100% and 35.8% area conditions and 600 trials for each of other conditions).

	**Area 100%**	**Area 35.8%**	**Area 7.5%**	**Area 2%**	**Area 0.47%**	**Area 0.12%**
**Area 100%**	-	t=0.9, p=1	t=2.4, p=0.2	t=3.5, p=0.01	t=4.6, p<0.001	t=5.2, p<0.001
**Area 35.8%**	-	-	t=1.5, p=1	t=2.6, p=0.21	t=3.6, p=0.01	t=4.4, p<0.001
**Area 7.5%**	-	-	-	t=1.0, p=1	t=2.1, p=0.63	t=2.9, p=0.08
**Area 2%**	-	-	-	-	t=1.0, p=1	t=1.9, p=1
**Area 0.47%**	-	-	-	-	-	t=0.8, p=1
**Area 0.12%**	-	-	-	-	-	-

Supplementary Table 2. Task with original contrast images. Marginal means and contrasts for each factor.

	SNR-contrast difference (standardized)	Target order presentation	Image repetition number
Estimated marginal means	= 0.57	First interval = 0.64<br>Second interval = 0.51	= 0.57
Contrasts	-	First interval = 0.06<br>Second interval = -0.06	-

Supplementary Table 3. Task with randomly inverted-contrast images. Marginal means and contrasts for each factor. In the last column, 0 indicates the condition in which both the target and the distractor have inverted contrast; 1 indicates the condition in which only the target has inverted contrast; 2 indicates the condition in which only the distractor has inverted contrast; 3 indicates the condition in which both the target and the distractor have original contrast.

	SNR-contrast difference (standardized)	Target order presentation	Image repetition number	Target contrast inversion
Estimated marginal means	= 0.53	First interval = 0.52<br>Second interval = 0.52	= 0.53	0 = 0.48<br>1 = 0.33<br>2 = 0.65<br>3 = 0.63
Contrasts	-	First interval = -0.001<br>Second interval = 0.001	-	0 = -0.05<br>1 = -0.19<br>2 = 0.13<br>3 = 0.10

Supplementary Table 4. Task with original contrast images in the trials with original-contrast target. Marginal means and contrasts for each factor.

	SNR-contrast difference (standardized)	Target order presentation	Image repetition number
Estimated marginal means	= 0.65	First interval = 0.67<br>Second interval = 0.62	= 0.65
Contrasts	-	First interval = 0.02<br>Second interval = -0.02	-
